# Resetting the epigenome: Methylation dynamics in cancer stem cells

**DOI:** 10.3389/fcell.2022.909424

**Published:** 2022-09-26

**Authors:** Aiendrila Roy, Swati Shree Padhi, Ibakordor Khyriem, Saket Nikose, Harsha Sankar S. H, Ruthrotha Selvi Bharathavikru

**Affiliations:** ^1^ Department of Biological Sciences, Indian Institute of Science Education and Research, Berhampur, Transit campus (Govt. ITI Building), Berhampur, Odisha, India; ^2^ EMBL, Rome, Italy; ^3^ Department of Biology, Indian Institute of Science Education and Research, Pune, Maharashtra, India

**Keywords:** chromatin, epigenetics, epitranscriptomic modification, cancer stem cells, RNA methylation, readers

## Abstract

The molecular mechanisms that regulate stem cell pluripotency and differentiation has shown the crucial role that methylation plays in this process. DNA methylation has been shown to be important in the context of developmental pathways, and the role of histone methylation in establishment of the bivalent state of genes is equally important. Recent studies have shed light on the role of RNA methylation changes in stem cell biology. The dynamicity of these methylation changes not only regulates the effective maintenance of pluripotency or differentiation, but also provides an amenable platform for perturbation by cellular stress pathways that are inherent in immune responses such as inflammation or oncogenic programs involving cancer stem cells. We summarize the recent research on the role of methylation dynamics and how it is reset during differentiation and de-differentiation.

## Introduction

The field of ‘Epigenetics’ has led to a “paradigm shift” in several domains of biomedical research (Deichmann, 2016). Waddington proposed the “epigenetic landscape” (EL) model in 1940, depicting a series of developmental options that a differentiating cell in the embryo could choose from. Epigenetics is now defined as “mitotically and/or meiotically heritable alterations in gene function that cannot be explained by changes in the DNA sequence.” The pluripotency of the undifferentiated cell and the eventual development of specific cell types is heavily reliant on the coordinated action of hundreds of transcription factors that bind to particular DNA regions to activate or repress cell lineage specific gene transcription (Srivastava and DeWitt, 2016). This establishment phase most closely reflects what is regarded as Conrad Waddington’s description of epigenetics, namely the study of the mechanisms by which the genotype gives the developmental phenotype. The maintenance phase usually involves a plethora of non-DNA sequence-specific chromatin cofactors that accumulate and maintain chromatin states through multiple cell divisions and for extended periods of time—sometimes even in the absence of the initial transcription factors (Schuettengruber et al., 2017).

### Methylation dynamics in stem cells

The stem cells have been excellent cellular models to understand the molecular mechanisms of epigenetics. Stem cells are capable of self-renewal and differentiation to all three lineages, and can be classified as follows: a) Naïve stem cells (derived from the zygote of the mammalian embryo, capable of self-renewal and unrestricted differentiation potential), b) Primed stem cells/Epiblast stem cells (EpiSCs) (that originate from the zygotic stage immediately after maternal redetermination post implantation, capable of self-renewal but have a more lineage restricted differentiation potential, c) Embryonic stem cells (ESCs) (derived from the inner cell mass of the blastocyst, capable of self-renewal and multi-lineage differentiation potential, d) Adult stem cells (ASCs), found in adult tissues and organs within their respective niche responsible for maintaining tissue homeostasis, repair and regeneration. These stem cells remain in a quiescent state till activation by a signal like cell damage, and capable of self-renewal and multi-lineage differentiation potential, e) Cancer stem cells (CSCs) that are derived from the dedifferentiation of cancer cells or from the malignant transformation of normal stem cells. These cells like any other stem cells have self-renewal abilities and multi-lineage differentiation potential and play a major role in the prognosis of the disease (Zhou and Zhang, 2008; Harikumar and Meshorer et al., 2015; Morena et al., 2018).

**FIGURE 1:**
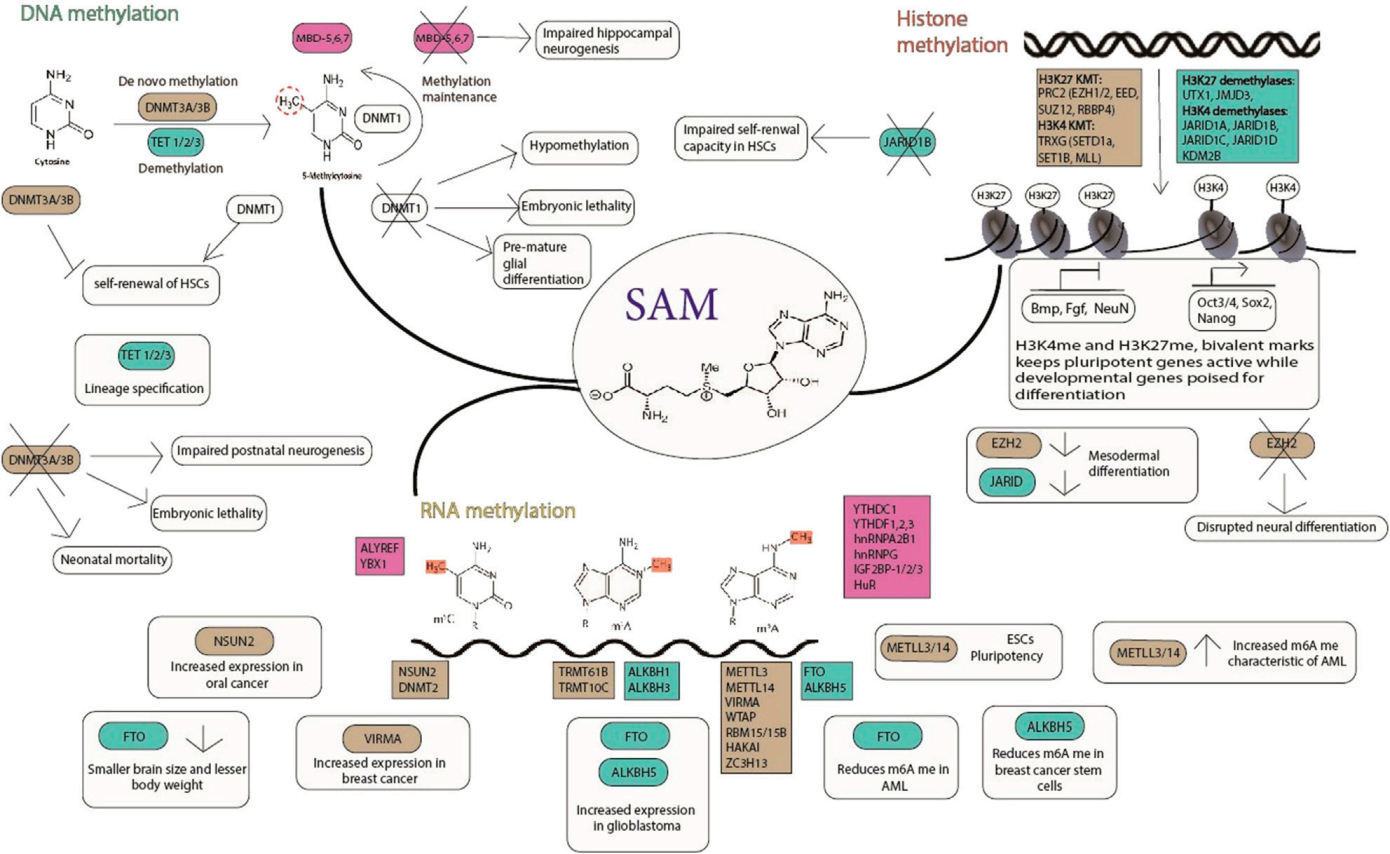
Methylation dynamics in stem cells; the readers (pink), writers (brown), and erasers (green) are indicated. SAM (S-adenosyl methionine) is the common methyl donor for histone, DNA and RNA methylation. The writers, erasers and readers play important roles in maintaining pluripotency and lineage commitment of stem cells. Their role in physiology and pathophysiology is depicted in the figure.

These unique characteristics of a stem cell are regulated by molecular mechanisms that involve transcription factors, signalling pathways, epigenetics and epitranscriptomics. Transcription factors such as Oct3/4, Sox2, c-Myc and Nanog bind to their target genes and regulate their expression (Harikumar and Meshorer et al., 2015). Many signalling pathways such as the JAK/STAT, PI(3)K, MAPK, Wnt, Notch, Smad and FGF pathways play major roles in regulating stemness. The epigenome dynamics contributes to the regulation of stemness which includes biochemical modification of DNA, RNA, histone proteins, and chromatin. These modifications are carried out by specific enzymes where the “writer” and “eraser” proteins catalyse the addition and removal of the modifications respectively, while other proteins called “reader” proteins specifically recognize these modifications (Figure 1).

**DNA Methylation:** A family of DNA methyltransferases (DNMTs), catalyzes the Methylation of cytosine’s fifth carbon position, leading to 5-methylcytosine (5 mC) formation. DNMT1 copies existing methylation patterns for inheritance during DNA replication, while DNMT3A and DNMT3B act as *de novo* methyltransferases to create new methylation patterns (Bestor, 2000). A group of methyl-CpG-binding proteins acts as readers, interpreting the 5 mC signal and mediating its role. While DNA methylation can be ‘passively diluted’ by cell division, mechanisms for enzymatic DNA methylation removal have been recently discovered. The ten eleven translocation 1 (TET1) enzymes, catalyzes the conversion of 5mC to 5hydroxymethylcytosine (5hmC) (Tahiliani et al., 2009). Following that, three TET family proteins were discovered to be able to oxidize 5hmC to 5formylcytosine (5 fC) and then to 5carboxylcytosine (5caC) (Ito et al., 2010; He et al., 2011; Ito et al., 2011). In addition, the deaminases activation induced cytidine deaminase (AID; also known as AICDA) and apolipoprotein B mRNA editing enzyme catalytic polypeptides (APOBECs) can convert 5hmC to 5-hydroxymethyluracil (5hmU). To complete the active DNA demethylation process, thymine DNA glycosylase excises all of these derivatives and replaces them with an unmodified cytosine through the base-excision repair (BER) pathway (Wu and Zhang, 2014).

**Histone methylation and demethylation:** Histone methylation is a dynamic process that plays important functions in differentiation and development (Eissenberg and Shilatifard, 2010). Basic residues like lysine and arginine undergo methylation and can have several methylations on their side chains (Greer and Shi, 2012). H3K4me3 and H3K27me3 are two histone modifications that have been linked to active and repressive transcription, respectively. A variety of lysine methyltransferases (KMTs) as writers and lysine demethylases as erasers can mediate dynamic methylation of lysine residues. Many proteins, including the well-known PcG repressive complex (PRC) and Trithorax active complex (TRXG), have KMT properties (Schwartz and Pirrotta, 2007; Greer and Shi, 2012). Methylation of H3K4, H3K36, and H3K79 is associated with transcriptional activation, and methylation of H3K9, H4K20, and H3K27 is related with transcriptional repression. Notably, “bivalent domains” which are thought to be crucial for maintaining pluripotency by silencing developmental genes in embryonic stem cells (ESCs) and keeping them ready for activation during developmental stage—are formed when large regions of H3K27 methylation co-occur with smaller regions of H3K4 methylation marks (Bernstein et al., 2006; Hu et al., 2013).

**RNA Methylation:** More than 100 post-transcriptionally modified ribonucleosides have been found in various forms of RNA (Jia et al., 2013). N6-methyladenosine (m6A) is a conserved modification found in most eukaryotic nuclear RNAs, as well as some viral RNAs replicating in the host nuclei (Carroll et al., 1990). m6A was discovered as an abundant nucleotide modification in eukaryotic messenger RNA in 1970 (Desrosiers et al., 1974). In global cellular RNAs, m6A is found in 0.1–0.4% of all adenosines and accounts for almost half of all methylated ribonucleotides. m6A modification is enriched in long internal exons, upstream of stop codons, and the 3′-UTR of mRNA, suggesting roles in translational regulation, affecting RNA binding protein affinities, or distinctive m6A derived transcriptome topology (Dominissini et al., 2012; Meyer et al., 2012; Batista et al., 2014). The discovery of proteins involved in m6A regulation, as well as their roles as “writers” (m6A methyltransferases), “erasers” (m6A demethylases), and “readers” (effectors recognizing m6A), has been one of the most significant achievements in this field of study (Lee et al., 2014), together facilitate various functional outcomes, including nuclear RNA export, splicing, mRNA stability, circRNA translation, miRNA biogenesis, and lncRNA metabolism (Roignant and Soller, 2017; Yang et al., 2017) thus regulating physiological and pathological events such as Yeast meiosis, plant development, immunoregulation obesity, and carcinogenesis (Wang et al., 2017; Wei et al., 2017).

### The epigenome in embryonic stem cells

Nucleosomes of stem cells show a higher level of modifications marks that are involved in active gene expression such as histone H3 lysine four trimethylation (H3K4me3), histone H4 lysine 9 and 14 acetylation (H3K9ac, H3K14ac). The two methyl modifications on H3K4 and H3K27 form a bivalent chromatin mark which is seen in the chromatin of stem cells. In stem cells, the highly conserved non-coding elements (HNCE) were found to be enriched with bivalent histone modifications, an active chromatin mark, H3K4me3 and a repressive chromatin mark, H3K27me3 (Bernstein, et al., 2006; Harikumar and Meshorer, 2015). These modifications are also abundant at promoter regions of genes that code for other factors required during development (Lessard and Crabtree, 2010). It is proposed that this bivalent chromatin mark resolves and there is activation of a few genes to regulate stemness while keeping other genes required for development poised for activation during development and cell differentiation (Bernstein et al., 2006; Lessard and Crabtree, 2010; Harikumar and Meshorer, 2015; Paranjpe and Veenstra, 2015). Recent studies have shown that many lineage-commitment genes have the bivalent mark and RNA polymerase II may be stalled at the promoters of these genes. During differentiation, the chromatin modifications are resolved into either an active or repressed state depending on the lineage commitment and these modifications can be newly established or maintained in differentiating cells (De Gobbi et al., 2011). Many early genes involved in the determination of the mesodermal lineage including various members of the GATA and Tbx families, Mixl1, and Brachyury, have bivalent domains in ES cells, supporting the notion that they are important early contributors (Pan et al., 2007). Histone arginine methylation has been shown to be important for pluripotency maintenance as well as lineage specification (Torres-Padilla et al., 2007; Selvi et al., 2015; Cui et al., 2017). Recent studies have shown that the RNA modifications have an important role in stem cell maintenance. The writer proteins are involved in controlling the expression of critical transcripts that are essential for stem cell self-renewal. m^6^A is shown to regulate molecular switches for differentiation and generation of EpiSCs, as well as in adult stem cells, like myeloid differentiation of hematopoietic stem cells (HSCs) (Morena et al., 2018).

### The epigenome during differentiation

The embryonic stem cells undergo multiple rounds of differentiation, resulting in multipotent or unipotent adult stem cell progenitors. Extrinsic differentiation signals and intrinsic pathways interact and tightly regulate how stem cells differentiate. The formation of neurons and other ectodermal lineage cell types, has been one of the most well studied differentiation pathways. The perturbation of DNA methylation, histone methylation or RNA methylation leads to defects in neurogenesis. In mice, a mutation in any of the three main *Dnmt* genes causes significant developmental defects and embryonic or early postnatal death (Li et al., 1992; Okano et al., 1999). Methyl-CpG binding domain protein 1 (MBD1) binds to hypermethylated CpG islands in gene promoter regions preferentially, and its absence impairs adult hippocampal neurogenesis and genomic stability *in vitro* (Zhao et al., 2003). PcG proteins and TRXG have also been linked to neurogenesis regulation. In embryonic cortical NPCs, deletion of Enhancer of zeste homologue two in PRC2 (Ezh2) causes a global loss of H3K27me3, derepression of a large number of neuronal genes, and disrupted neuronal differentiation (Pereira et al., 2010). The RNA demethylase FTO has been shown to be expressed in adult neural stem cells and neurons and exhibits dynamic expression during postnatal neurodevelopment.

The role of the epigenome on differentiation has also been well studied in the hematopoietic stem cells (HSC). *Hox* genes, critical for maintaining the balance between self-renewal and differentiation of HSC and progenitor cells are associated with bivalent domains in undifferentiated ESCs and its sequential expression during differentiation is regulated by PcG and TRXG proteins (Deng et al., 2013). Hematopoietic specific genes such as CD45, CD34 among others exhibited repressive DNA methylation marks prior to differentiation of the ESC which are subsequently lost upon differentiation correlating with gene expression (Suelves et al., 2016). DNMT3a and DNMT3b act to repress self-renewal genes in HSCs and their combined loss enhances self-renewal by activating β-catenin signalling (Sharma and Gurudutta, 2016). DNMT1 aids in efficient hematopoietic differentiation and is crucial for the progression of cells to multipotent progenitors to lineage-restricted myeloid and lymphoid progenitor cells. DNMT3b is responsible for the *de novo* methylation of hematopoietic genes during early embryogenesis (Suelves et al., 2016). Deletion of the histone demethylase JARID1b compromises the self-renewal capability of the HSCs (Sharma & Gurudutta, 2016). The RNA m6A modification writer METTL, has also been shown to be essential for the symmetric division of HSCs (Cheng et al., 2019).

### The epigenome in CSCs, during dedifferentiation

Cancer Stem Cells (CSCs) are a small subpopulation of cells within tumors, which are capable of self-renewal, differentiation, and tumorigenicity when transplanted into an animal host. CSCs can be distinguished from other cells within the tumor by differences in their cell division and gene expression (Rosen and Jordan, 2009). The first evidence for the presence of CSCs was shown in a study where leukemia initiating cell population from AML patients was identified based on the expression of (CD34+/CD38−) cell surface markers, by transplantation into severe combined immune-deficient (SCID) mice (Lapidot et al., 1994). The existence of Glioma stem cells (GSC) was first hypothesized in 2002, when it was considered to have migrated from the sub-ventricular niche. (Ignatova et al., 2002). It has now been shown that the genome-wide distribution of epigenetic signatures is associated with the differential programming of GSC and Neuronal Stem Cells (NSC) (Valor Luis and Hervás-Corpión 2020). CSCs are resistant to conventional chemotherapy or radiation treatment and can contribute to metastasis through the dedifferentiation process (Meirelles et al., 2012). High methylation can contribute to the self-renewing ability of CSCs during tumor progression (Muñoz et al., 2012). The epigenome modifications of CSCs play a major role in recurrence, metastasis, and therapeutic failure.

### Resetting the epigenome through methylation dynamics

The dynamicity of the methylation mark on DNA, histones or RNA serves as an important biochemical rheostat for regulating stem cell pluripotency and lineage commitment along with other regulatory factors (Berdasco and Esteller, 2011; Völker-Albert et al., 2020; Sun et al., 2021). The reversible nature of these modifications provide an easy and efficient modulatory node that is used by cancer stem cells (Vincent et al., 2019; Carvalho, 2020). The expression of transcription factors, signalling pathways and other regulatory proteins in stem cell biology are under the control of this reversible modification.

**FIGURE 2:**
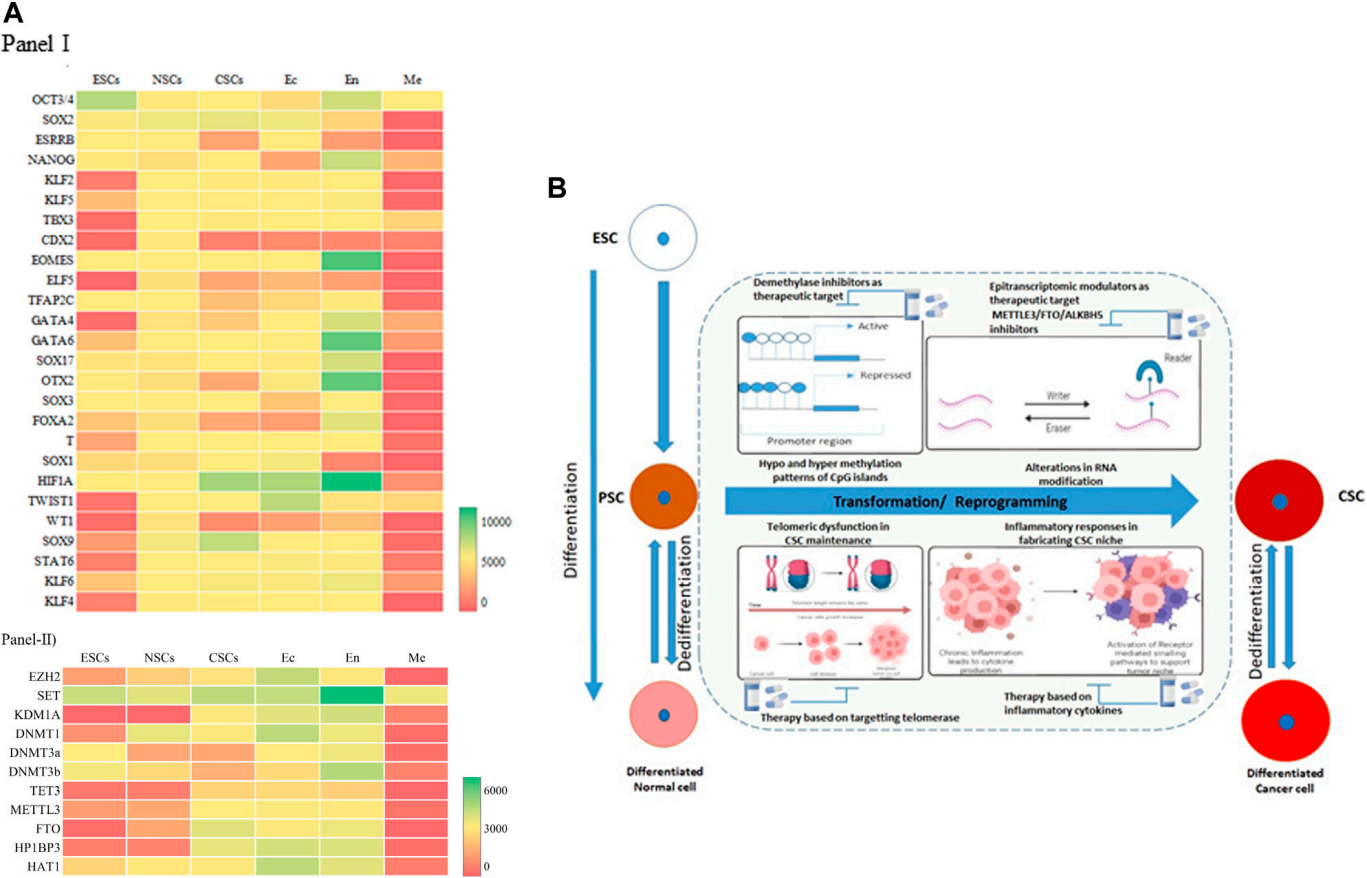
Resetting the Epigenome Dynamics in Differentiation and Dedifferentiation. **(A)** Heat map of Panel I, Transcription Factors (26) expression and Panel II modifiers (11) such as Histone methyltransferase/demethylase, DNMT/demethylase, RNA methyltransferase/demethylase in different cell types- embryonic stem cells (ESCs), neural stem cells (NSCs), cancer stem cells (CSCs), ectoderm (Ec), endoderm (En), mesoderm (Me). The red color indicates a lower expression level, and the green indicates a high expression level. Yellow represents intermediary expression levels. The intensity of the color indicates the expression level. Gene Expression Omnibus (GEO) database (https://www.ncbi.nlm.nih.gov/geo/) was used to gather the data for the six types of cells/tissues EMBRYONIC STEM CELLS (Datasets- GSE220881 and GSE775182), NEURAL STEM CELLS (Datasets- GSE380453 and GSE353904), CANCER STEM CELLS (Datasets- GSE433785 and GSE42906), ECTODERM (Datasets- GSE339037 and GSE1442418), ENDODERM (Datasets- GSE1080479, GSE5528310, and GSE2413511), MESODERM (Datasets- GSE18216112 and GSE11477613). Excel software was used to plot the heatmap. The selected data is converted into the heat map using conditional formatting (color scales). **(B)** A combination of various oncogenic events triggers transition of pluripotent stem cells and differentiated cells to cancer stem cells. Differential methylation at the CpG islands triggers oncogenes and transcription factors leading to emergence of tumor heterogeneity and CSCs. Alterations in global epitranscriptomic profile also regulate the reprogramming or dedifferentiation events. Cytokine and Interleukins acts in paracrine manner leading to cancer inflammation and crafts a niche for emergence of stem like cells by activating downstream signalling pathways. Telomere associated quiescence supports the stem like cellsin the tumor micro environment elevating their self-renewal capacity.

A meta analysis of the available datasets was done to assess the changes that occur during these stages, as shown in Figure 2A. The transcription factors Oct3/4, Sox2, and Nanog expression are upregulated in ESCs because they are the core transcription factors in maintaining the pluripotency of the embryonic stem cells (Boyer et al., 2005). At the same time, Elf5, Gata4, Wt1, Stat6, Klf2, Tbx3, Cdx2, etc., are downregulated in ESCs. In Neural Stem Cells (NSCs) (NSCs), almost all of the TFs have average expression levels (Figure 2A, Panel I), with Sox2 at the highest level of expression. The cancer stem cells (CSCs) in gliomas, that would have undergone a dedifferentiation, show a very different expression level compared to the NSCs. Sox2, Sox9, and HIF1A show increased expression, whereas Cdx2, Esrrb, Wt1, etc., show decreased expression in CSCs. These expression levels could be the markers of cancer stem cells (Zhao et al., 2017). On comparing the three germ layers (Ectoderm, Endoderm, and Mesoderm), the expression level of TFs changes significantly, especially in the mesodermal lineage. The cells or tissues showing the elevated expression of the Eomes, Hif1a, Gata6, Gata4, Sox17, Otx2, etc., can be identified as an endodermal lineage. In addition to this, there is an expression of pluripotency factors such as Oct3/4 and Nanog. In ectodermal cells, we see the upregulated expression of Hif1a, Twist1, Sox2. Interestingly, the expression profile of ectodermal cells is somewhat similar to the CSCs.

The epigenome modifiers such as Histone methyltransferases/demethylases, DNMTs/demethylases, RNA methyltransferases/demethylases also have dynamic expressions in the different cell types (Rwigemera et al., 2021). In ESCs, most of the transcription factors have moderate expression. SET has a higher expression level as opposed to the KDM1A, TET3, and FTO (Chung and Sidhu, 2008). NSCs also follow the same trends as ESCs (Figure 2A, Panel II). Ectodermal cells have higher expression levels of epigenome modifiers. Most epigenome modifiers have lower expression in the mesodermal cells except SET, KDM1A, DNMT3b, and HAT1. In CSCs, all the modifiers express moderately, except SET, FTO, and HP1BP3. This suggests an intermediary state of gene expression in the CSCs, where additional environmental factors can then come into play and facilitate tumour manifestation. It has been shown that Glioma stem cells (GSC), once formed, are also regulated by various signalling pathways, coordinated by epigenetic reprogramming. GSCs are reported to overexpress histone demethylase KDM4C, which removes H3K9me3 from Wnt target genes, promoting Wnt/Signalling Pathway and thereby stem cell maintenance (Chen et al., 2020; Kumar et al., 2022). Epigenetic regulators maintain tumoral hierarchy through two mechanisms, either through inhibition of self-renewal property of cancer cells thereby maintaining heterogeneity, or by facilitating CSCs in evading differentiation and maintenance of stem cell phenotype (Wainwright and Scaffidi, 2017; Valor Luis and Hervás-Corpión, 2020; Tao et al., 2022). RNA Methyltransferase, METTL3-mediated RNA stabilization positively regulates major signalling pathways such as Notch, NFκB, Wnt, c-Myc, TGFβ, involved in cancer stem cell maintenance and proliferation in several cancers including Glioma and Leukemia maintenance and tumorigenesis implying its oncogenic role (Visvanathan et al., 2018).

In this context, the inflammatory pathway has been shown to be recognized as a major component of tumorigenesis in various cancers. Solid tumors are also associated with Tumor Associated Macrophages (TAM) which constitute various immune infiltrating cells. These TAMs and stromal cells secrete cytokines such as Interleukin 1(IL1), IL6 and TNFα acts in paracrine fashion for sustenance and reprogramming of CSCs, by altering epigenetic mechanisms and thereby regulating transduction pathways such as NFκB, STAT3 and SMADs. (Biswas et al., 2013). These inflammatory pathways interconnect to form molecular regulatory circuits in resetting the networks for maintaining CSCs (Liu et al., 2021). Chronic inflammation can initiate DNA damage response in preneoplastic lesions, leading to telomere loss (Shay and Wright 2010). This triggers segregational defects, activation of telomerase and setting in of genomic instability, one of the major hallmarks of cancer. Patient derived CSCs in glioma have demonstrated shortened telomeres along with telomerase expression indicating the fact that GSCs are not quiescent and have the capacity for aberrant self-renewal properties (Koeneman et al., 1998). A summary of the alterations in reprogramming/transformation and de-differentiation is represented in Figure 2B. Inflammation regulates the acquisition and maintenance of the cancer stem cell phenotype by stimulating epithelial mesenchymal transitions. Many inflammatory factors like IL-1β, TGF-β, IL-6 can regulate the DNA methylation patterns that induce cancer initiation and progression in cancers such as gastric cancer, ovarian cancer, and liver cancer (Liu et al., 2021). The exact mechanisms of how the epigenome dynamics facilitates this process warrants further investigation which will provide useful therapeutic intervention prospects.
